# Eumelanin Graphene-Like Integration: The Impact on Physical Properties and Electrical Conductivity

**DOI:** 10.3389/fchem.2019.00121

**Published:** 2019-03-18

**Authors:** Roberto Di Capua, Valentina Gargiulo, Michela Alfè, Gabriella Maria De Luca, Tomáš Skála, Gregor Mali, Alessandro Pezzella

**Affiliations:** ^1^Department of Physics “E. Pancini”, University of Naples “Federico II” and Superconducting and Other Innovative Materials and Devices Institute (SPIN), CNR, Naples, Italy; ^2^Institute for Research on Combustion (IRC), CNR, Naples, Italy; ^3^Faculty of Mathematics and Physics, Charles University, Prague, Czechia; ^4^Department of Inorganic Chemistry and Technology, National Institute of Chemistry, Ljubljana, Slovenia; ^5^Institute for Polymers, Composites and Biomaterials (IPCB), CNR, Pozzuoli, Italy

**Keywords:** melanin, eumelanin, graphene-like layers, hybrid materials, solid state nuclear magnetic resonance, synchrotron radiation, spectroscopical characterization

## Abstract

The recent development of eumelanin pigment-based blends integrating “classical” organic conducting materials is expanding the scope of eumelanin in bioelectronics. Beyond the achievement of high conductivity level, another major goal lays in the knowledge and feasible control of structure/properties relationship. We systematically investigated different hybrid materials prepared by *in situ* polymerization of the eumelanin precursor 5,6-dihydroxyindole (DHI) in presence of various amounts of graphene-like layers. Spectroscopic studies performed by solid state nuclear magnetic resonance (ss-NMR), x-ray photoemission, and absorption spectroscopies gave a strong indication of the direct impact that the integration of graphene-like layers into the nascent polymerized DHI-based eumelanin has on the structural organization of the pigment itself, while infrared, and photoemission spectroscopies indicated the occurrence of negligible changes as concerns the chemical units. A tighter packing of the constituent units could represent a strong factor responsible for the observed improved electrical conductivity of the hybrid materials, and could be possible exploited as a tool for electrical conductivity tuning.

## Introduction

Eumelanin belongs to the melanin pigments family and it is the pigment type mostly found in bacteria, fungi, plants, animals, and extinct organisms. It is involved in ultraviolet (UV) protection, detoxification, metal binding, and structural coloration (D'Ischia et al., 2013). Eumelanin is biosynthesized in the melanocytes from tyrosine through a series of enzyme-catalyzed reactions: the eumelanin precursors–5,6-dihydroxyindole (DHI), 5,6-dihydroxyindole-2-carboxylic acid (DHICA) and their oxidized forms—are cross-linked into the polymer chain through chemical bonds or physical interactions (D'Ischia et al., 2013).

The possibility to synthesize the eumelanin under biomimetic conditions is attracting deep interest and in the meantime is also expanding eumelanin potential relevance for applications in bioelectronics. In particular, DHI derived eumelanins have been proven to be highly biocompatible (Bettinger et al., 2009; Gargiulo et al., 2015), a feature that, together with their water dependent hybrid ionic-electronic conductor nature (Rettenwander et al., 2014; Gargiulo et al., 2015; Wunsche et al., 2015) linked to the specific functionalization of the pigment (Jastrzebska et al., 1995; Eom et al., 2016), makes such pigments of enormous potential interest for biosensing applications. In particular, the electrical (ionic as well as electronic) conductivity of these pigments suggested their application as biointerface capable to allow wealthy cell culture and at the same time to transfer electrical signaling between the cell culture and underlying electronic devices (D'Ischia et al., 2013; Barra et al., 2015).

A major limiting factor for the actual implementation of eumelanin-based electronic devices comes from the low absolute value of conductivity of this pigment, and from the fact that the actual conduction mechanisms are still matter of debate (Meredith et al., 2013; Mostert et al., 2013; Sheliakina et al., 2018). Different strategies are appearing in order to circumvent the poor conductivity limitation, in order to exploit the biocompatibility and the favorable chemical properties of eumelanin (Meredith et al., 2013; Gargiulo et al., 2015). In this context, by the integration of conductive graphene-like (GL) layers within the eumelanin pigment (EU), we recently explored the possibility to synthesize a novel organic/organic hybrid material (EUGL). EUGL was confirmed to be widely biocompatible, and to strongly enhance the intrinsic poor electrical transport of eumelanin (Gargiulo et al., 2015; Papari et al., 2017). It was speculated that this conductivity increase has a contribution from the improved large-scale homogeneity of the samples associated with a better connection between uniformly distributed grains of the two starting materials and the formation of percolating paths of GL layers inside the material. However, it is hard to clearly correlate the observed macroscopic transport behaviors to microscopic mechanisms, since the eumelanin chemical structure is still not completely known (Huang et al., 2018). Some studies suggested that eumelanin could be regarded as a very high-molecular-weight polymer, with fundamental units randomly linked (chemical disorder) (McGinness et al., 1974; Tran et al., 2006). Morphological and x-ray diffraction experiments (Cheng et al., 1994; Clancy and Simon, 2001; Liu and Simon, 2003), instead, indicated the presence of a supra-molecular order, based on the concept of hierarchical assembly from basic monomers to larger aggregates (Clancy and Simon, 2001). Chemical studies on water-soluble eumelanin and on precipitation phenomena were interpreted ascribing a relevant role to the aggregation-dependent intermolecular perturbation of the π-electron systems (Pezzella et al., 2009; Arzillo et al., 2012; Ascione et al., 2013). In addition to the difficulties in clarifying the transport mechanisms in pure eumelanin, in the case of hybrid compounds it is hard to assign suitable conduction models without direct information on local organization and on the role of functional groups.

The achieved result with the novel organic/organic hybrid material EUGL and the need to get a better understanding of this system spurred the interest toward the investigation of structural factors associated to the electronic conductivity improvement in order to gain valuable tools for a feasible control over the macroscopic properties. Here we present a detailed integrated characterization of three EUGL hybrid compounds containing different amounts of GL layers combining basic laboratory characterizations with experiments of solid state nuclear magnetic resonance (ss-NMR) and advanced photoemission techniques by synchrotron radiation, with the aim of focusing on the modification of molecular packing induced by the integration of the eumelanin pigment with GL layers.

## Materials and Methods

### Materials

Reagents and solvents (analytical grade) were purchased from Sigma Aldrich and used without further purification. Carbon black (CB, furnace black, N110 type, 15–20 nm primary particles diameter, specific BET area 139 m^2^ g^−1^) was purchased from Sid Richardson Carbon Co.

### Sample Preparation

GL layers were produced following a two-step oxidation/chemical reduction approach previously reported (Alfè et al., 2012, 2014a,b, 2015). The applied method is briefly described in the following: CB powder (0.5 g) was treated with nitric acid (10 mL of HNO_3_ 67 % v/v) at 100°C and after 90 h under stirring and reflux the oxidized carbonaceous material, named GL-ox, was recovered by centrifugation, washed with distilled water until the neutrality was reached, dried at 100°C and stored. An amount of GL-ox (0.06 g) was then dispersed in distilled water (60 mL to reach 1 mg mL^−1^ as mass concentration) and treated for 24 h with hydrazine hydrate (N_2_H_4·_× H_2_O, 1.35 mL) at 100°C under reflux. After that time, the black suspension was cooled at room temperature and diluted nitric acid (HNO_3_ 4 M) was dropwise added to neutralize the excess of hydrazine. The decrease of the pH solution allowed the precipitation of the GL layers as a black solid. GL layers were then recovered by centrifugation, washed with distilled water, and recovered again by centrifugation twice. At the end of the purification process, the material was stored as aqueous suspension (mass concentration 1 mg mL^−1^, pH 3.5) and as powder for bulk characterization after drying at 100°C.

DHI was prepared according to a procedure previously described (Edge et al., 2006; D'Ischia et al., 2013). Eumelanin was synthetized under biomimetic conditions through DHI oxidative polymerization (Edge et al., 2006; Gargiulo et al., 2015; Papari et al., 2017). A proper amount of DHI (20 mg) was dissolved in methanol (1 mL to reach a mass concentration of 20 mg mL^−1^) by ultrasonic agitation and the resulting solution was stirred for 10 min. Then an ammonia solution (28% v/v in water) was dropwise added to adjust the solution pH to 8 and to allow the auto-oxidization and polymerization of indole molecules; the pH of the mixture is a critical parameter in this process and a fine and accurate control is required. After 30 min, diluted acetic acid (1 M) was dropwise added to quench the reaction by reaching a solution pH around 4. A part of the reaction mixture was stored as it is while another part was dried at 100°C and the resulting powder stored as EU.

**Figure 1:**
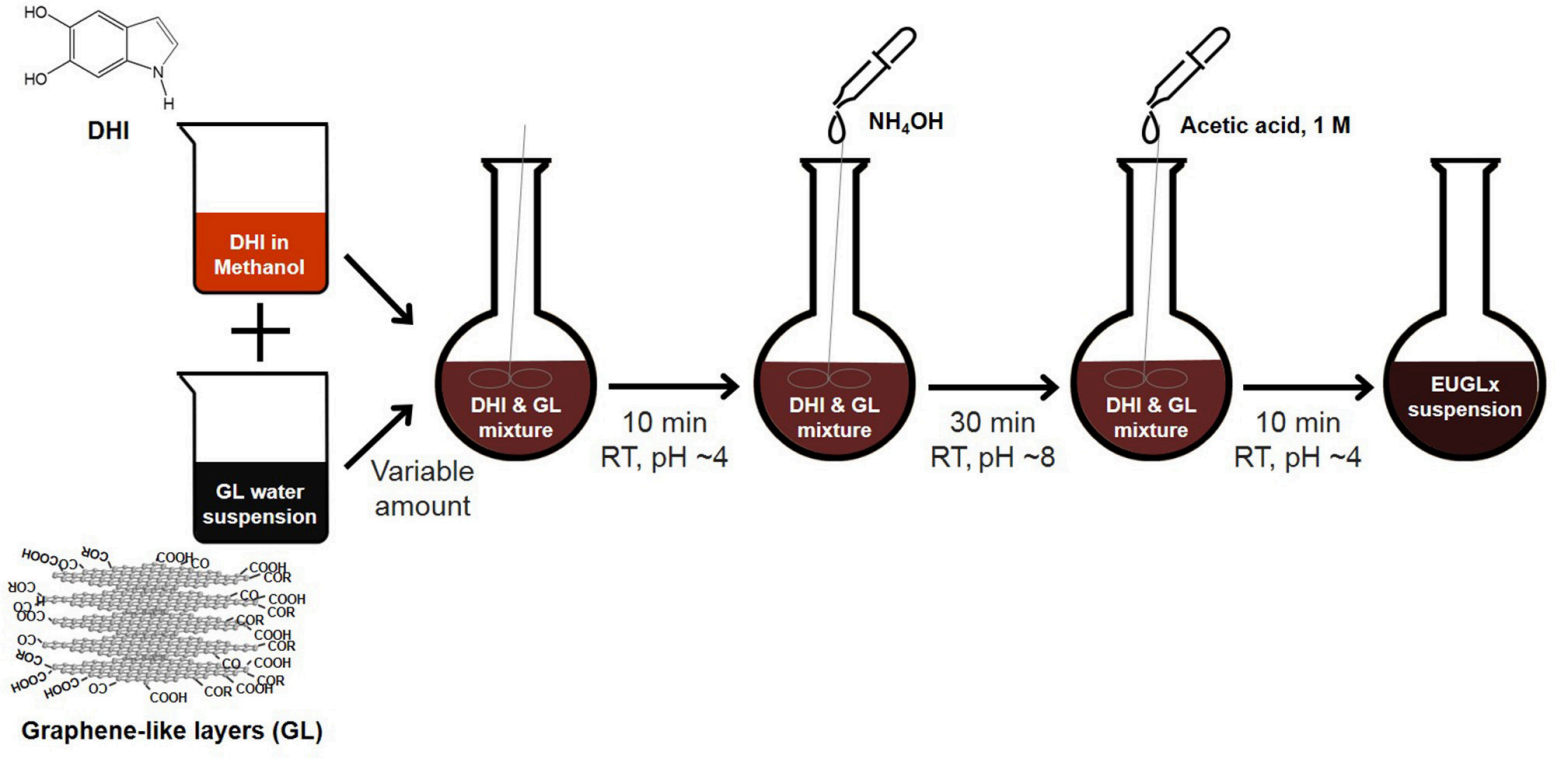
Schematic diagram of the synthetic procedure adopted for the preparation of the EUGLs hybrids.

The hybrid materials were prepared adapting the procedure previously described (Gargiulo et al., 2015; Papari et al., 2017). The integration of GL was achieved via *in-situ* exposure of GL layers to the nascent polymer under mildly alkaline conditions. Briefly, for the preparation of each hybrid a proper volume of aqueous suspension of GL layers (1 mg mL^−1^) was added to a DHI methanol solution (20 mg mL^−1^) before the addition of ammonia solution to induce the DHI oxidation and polymerization. The amount of GL layers was varied to prepare hybrids with different EU/GL mass ratios (Table 1). The work-up of the reaction was the same as described for the preparation of pure eumelanin. A sketch of the synthetic procedure is reported in Figure 1. Each suspension was in part stored and in part it was dried in an oven at 100°C and the resulting powdered material stored for bulk analysis. After drying, all EUGL hybrids resulted insoluble in water.

**Table 1:** Elemental composition of the investigated samples.

**Sample label**	**% GL content**	**% EU content**	**Elemental composition**			
			**C wt.%**	**H wt.%**	**N wt.%**	**O wt.%**
GL	100	0	52.9	1.4	6.1	39.6
EU	0	100	52.5	3.3	7.7	36.6
EUGL1	33	67	52.7	3.2	9.5	34.5
EUGL2	50	50	54.4	3.9	9.4	32.3
EUGL3	67	33	56.3	2.8	8.2	32.7

A sample simulating the composition of EUGL3 was prepared by mixing polymerized EU and GL both as powders in the following amounts: 6.6 mg of GL and 3.4 mg of EU. The result of such physical mixture was named EU+GL3.

### Methods

The elemental composition of the samples in terms of carbon, hydrogen, and nitrogen contents was measured by ultimate analysis performed with a CHN 628 LECO elemental analyzer following the ASTM E870 procedure and using EDTA as standard. For each sample two measurements were performed and the values are reported as average with a maximum relative error around 0.7%. Oxygen content was evaluated by difference.

Fourier Transform Infrared (FTIR) spectra were acquired on pellets prepared by pressing at 10 ton for 10 min solid dispersions containing the powdered materials mixed with KBr (mass concentration 0.5–0.8 wt.%). FTIR spectra in the 3,400–600 cm^−1^ range were acquired in the transmittance mode using a 5,700 Nicolet spectrophotometer.

Thermogravimetric analyses (TGA) were performed on a Perkin–Elmer Pyris 1 thermogravimetric analyzer. The thermal stability of the samples was evaluated under an oxidative environment (air, 30 mL min^−1^) by heating each material from 50°C up to 800°C at a rate of 10°C min^−1^.

Each reaction mixture was used without any further treatment for the preparation of the samples for AFM, Scanning electron microscopy (SEM), and IV-measurements. The aqueous mixture was drop-casted onto mica (AFM imaging) or glass plates (SEM imaging and IV measurements) and allowed to dry at room temperature in air for 48 h.

The AFM images were acquired on an XE100 Park instrument operating in the non-contact mode (amplitude modulation, silicon nitride cantilever from Nanosensor) at room temperature and in ambient conditions.

Scanning electron microscopy (SEM) measurements were performed on a FEI Inspect TMS50 Scanning Electron Microscope. Scanning was performed on samples previously sputter coated with a thin layer of gold to avoid charging.

A four-contacts (van der Pauw, 1958) configuration was employed to estimate the dc electrical conductivity of the samples, drop-casted on glass substrates. Voltage supply and current measurements were provided by a Keithley Electrometer 6517A and a Keithley pico-amperemeter 6,485, respectively. The drop-casted samples after drying were contacted by small droplets of silver-based conducting glue, realizing a square-shaped four-contacts geometry (according to the van der Pauw requirements). The electrical measurements were performed in air at a relative humidity (RU) of about 25%.

The solid state Nuclear Magnetic Resonance (ss-NMR) measurements were performed at the Slovenian NMR Center in Ljubljana. ^1^H and ^13^C magic-angle spinning (MAS), and ^1^H-^13^C cross polarization magic-angle spinning (CPMAS) spectra were recorded on a Varian 600 MHz VNMRS spectrometer equipped with a 3.2 mm HX MAS probe. Larmor frequencies for ^1^H and ^13^C nuclei were 599.58 and 150.77 MHz, respectively. In the ^1^H MAS NMR experiment samples were spun with rotation frequency of 20 kHz, protons were excited with a 90-degree pulse of 2.3 μs, repetition delay was 10 s, and 16 scans were co-added. In the ^1^H-^13^C CPMAS experiment the polarization was transferred from protons to carbon nuclei in a 0.8 ms CP block, repetition delay between scans was 0.1 s, and the number of scans was 150,000. Sample rotation frequency was 16 kHz. During acquisition of the carbon signals high-power proton decoupling was employed. The ^1^H and ^13^C chemical shift axes were referenced to tetramethylsilane.

X-ray photoemission spectroscopy (XPS) and resonant photoemission spectroscopy (ResPES) experiments were performed at the Materials Science Beamline (MSB), Elettra synchrotron light facility in Trieste, Italy. The MSB, with a bending magnet source, provides synchrotron light in the energy range 21–1,000 eV. The basic set-up at MSB consists in a chamber including a dual Mg/Al X-ray source with a base pressure of 2 × 10^−10^ mbar and a SPECS Phoibos 150 hemispherical energy analyzer.

Al Kα radiation (1486.6 eV) was used to measure the core levels of C 1s, N 1s, O 1s. The incident photon energies were 410, 475, and 630 eV, respectively, with total resolutions of 350, 500, and 700 meV, respectively. The core level spectra were acquired at constant pass energy and at an emission angle of 0° with respect to the surface normal. The binding energy positions of the features in the XPS spectra were aligned with those measured by the laboratory source, for which the binding energy was referenced to the Fermi edge of Au(100) (EB = 0).

The reproducibility of the spectroscopical and electrical characterization data was assessed by measuring at least two fresh aliquots of selected samples.

## Results and Discussion

In Table 1, all samples analyzed in this work are listed with their label. For each sample, the measured elemental composition is reported together with the percentages of GL and EU established on a ponderal basis starting from the milligrams of the GL and the EU precursor used for the preparation of each sample.

The compositions of the parent materials (EU and GL) are quite similar (the elemental composition of DHI-based eumelanin estimated in this work is in accordance with that reported by a similar material in Glass et al. (2012); Saini and Melo (2015)), apart a considerably lower hydrogen amount in GL. As a consequence, the compositions of the hybrid materials do not exhibit large difference among them and with respect to EU and GL.

The measured values of C, H, and N in the hybrids indicated a complete incorporation of the GL layers into the hybrids. As a matter of fact, a good accordance between the measured and the “theoretical” percentages of each element content was found. Related data and trends are provided as (Figure S1).

**Figure 2:**
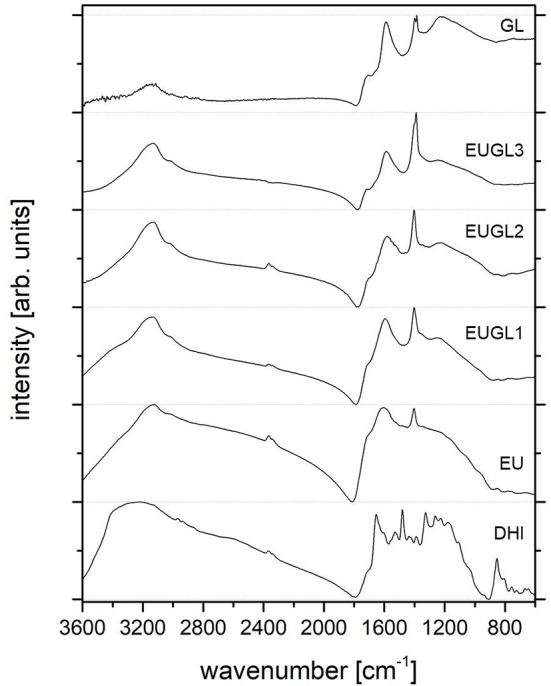
FTIR spectra of all the investigated samples. Height normalized spectra have been vertically shifted for clarity.

The FTIR spectra of the materials are reported in Figure 2. The spectra are characterized by broad peaks, confirming the chemical heterogeneity of the investigated samples: GL, EU, and EUGL samples exhibit complex vibrational spectra with many overlapping modes, making an accurate vibrational mode assignment very challenging.

As previously reported (Alfè et al., 2012; Gargiulo et al., 2015), the main spectral features of the GL layers FTIR spectrum are: a broad band in the 3,000–3,600 cm^−1^ range ascribable to stretching vibrations of O–H in carboxylic and phenolic groups and possibly adsorbed H_2_O, and to stretching bands (at lower wavenumbers) of N–H in hydrazones; a band at 1,710 cm^−1^ ascribable to C=O stretching vibrations from carbonyl and carboxyl groups, anhydrides, lactones, single ketones, and quinones; a band around 1,590 cm^−1^ due to skeletal vibrations of the π-conjugated graphitic domains; overlapping bands between 1,500 and 1,100 cm^−1^ due to bending modes of aromatic moieties; an intense band at 1,384 cm^−1^ ascribable to nitric groups.

The EU FTIR spectrum is characterized by a broad band at 3,150 cm^−1^ ascribable mainly to C-H stretching bands of five-membered N-heterocycles (pyrrole) (Cataliotti and Paliani, 1976; Snavely et al., 1992) with a shoulder at 3,000 cm^−1^ ascribable to aromatic C–H stretching modes (Silverstein et al., 2008). In the region between 1,700 and 1,000 cm^−1^, the broad band at 1,600 cm^−1^ can be assigned to C=C aromatic/pyrrole ring stretching vibrations, while its shoulder at 1,700 cm^−1^ corresponds to C=O stretching in quinone and/or ketone moieties. The presence of C=O stretching signal as a shoulder of the intense band at 1,600 cm^−1^ and not as distinct peak suggests that the content of indole/quinone units is not relevant (Glass et al., 2012). Apart from the intense peak at 1,400 cm^−1^ ascribable to pyrrole ring stretching [C–O, C–C, and C–N in plane modes (Centeno and Shamir, 2008)], the overlapping bands between 1,500 and 1,000 cm^−1^ going to 1,000 cm^−1^ generate a broad profile and no accurate assignment is possible.

The FTIR spectra of all the EUGLs are quite similar to that of EU, yet for the hybrid richest in GL (EUGL3). It is worth noting that, even if GL is very rich in C=O containing groups (Alfè et al., 2015), the peak due to C=O stretching modes at 1,700 cm^−1^ is present only as a shoulder (of increasing sharpness as GL content increases) of the intense peak at 1,600 cm^−1^ related to C=C aromatic/pyrrole ring stretching vibrations. The peak at 3,150 cm^−1^ due to aromatic pyrrolic C–H stretching, and the pyrrole ring stretching peak at 1,400 cm^−1^ are also present in the spectra of all the hybrid compounds. Interestingly, the larger spectral weight of the latter two peaks in EUGLs than in EU, despite the lack of such features in GL, suggests the presence of specific chemical functionalities near to the surface that are partially covered in the pure EU.

The presence of the typical signatures of eumelanin in all the EUGLs FTIR spectra indicates the efficacy of the developed procedure to achieve the merging of the two starting materials. Moreover, by comparing the FTIR spectrum of the starting indole (DHI) with those of the hybrids, the actual formation of eumelanin in the presence of GL layers is proved.

The thermal stability of the samples was investigated by measuring the mass loss of the samples upon heating up to 800°C in an oxidative environment (Figure S2) in a thermogravimetric apparatus. The thermal profiles of EUGLs exhibit features similar to those of parent materials: two main weight losses at 200°C and 550°C, the latter corresponding to the EUGL burn-off. The absence of a sharp weight loss at around 550°C, observed for neat GL layers, in the TG profiles of all hybrids suggests an intimate contact between the hybrid components (EU and GL layers).

**Figure 3:**
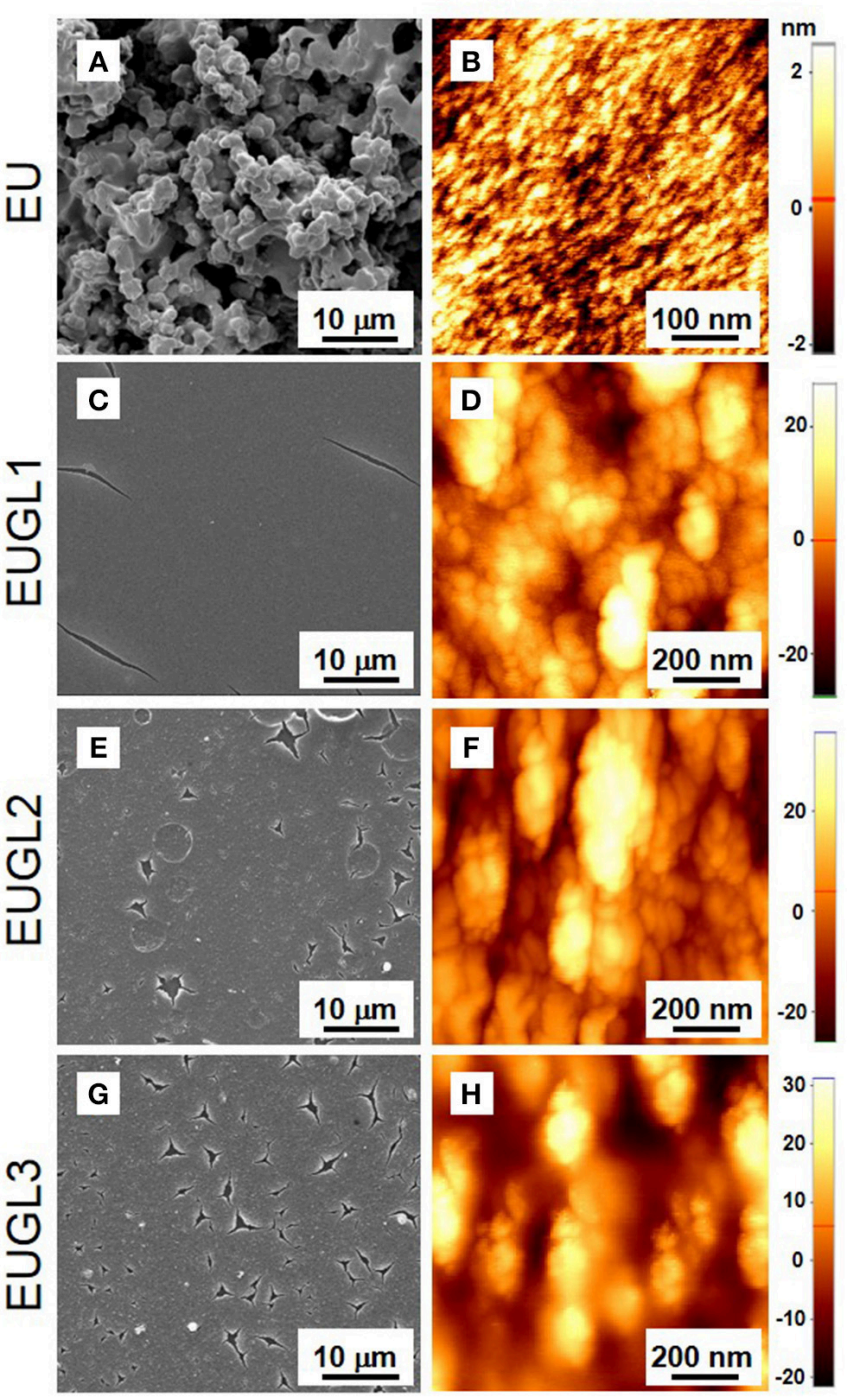
SEM **(A,C,E,G)** and AFM **(B,D,F,H)** images of EU, EUGL1, EUGL2, and EUGL3.

The morphology of the samples was investigated by SEM and AFM (Figure 3). Overall the SEM imaging revealed that the EUGLs samples present a wide homogeneity over a large scale with respect to the granular feature exhibited by EU. Each EU large (macroscopic) agglomerate exhibited a surface characterized by a fine granularity with height of the order of few nm (Figure 3B). On the other hand, EUGLs AFM imaging (Figures 3D,F,H) showed microscopic grains larger and higher than in EU and slightly increasing with the GL amount, a possible consequence of the different packing discussed in the following.

**Figure 4:**
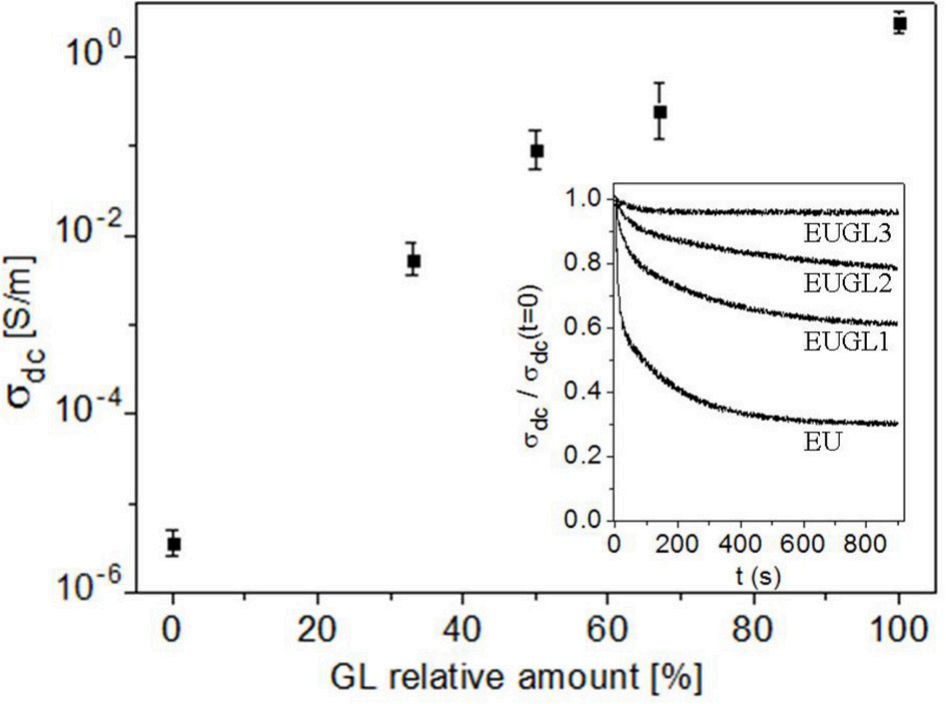
σ_dc_ electrical conductivity of the investigated samples (identified by the GL amount); in the inset, the time-decay of σ_dc_ for EU and EUGLs samples is reported. Measurements have been performed at controlled condition of relative humidity (RU) of about 25%.

Four-probe measurements performed in the classical van der Pauw configuration (van der Pauw, 1958) provided dc electrical conductivity σ_dc_ of the investigated materials. No deviation from the ohmic behavior was observed in the explored voltage range. The estimated dc electrical conductivities are plotted in Figure 4.

As expected, σ_dc_ monotonically increases with the GL content, from the value of 5 μS/m in eumelanin to the GL value of 2.5 S/m. This result confirms what already reported in (Gargiulo et al., 2015), as well as the observed time-decay of σ_dc_ on EU and on the hybrids, shown in the inset of Figure 4. On EU, EUGL1, and EUGL2, the σ_dc_ decay appears as the overlapping of two exponential decays on different time scale. The presence of two different time scales can be interpreted as related to both ionic and electronic contributions (Cicco et al., 2015; Gargiulo et al., 2015; Wunsche et al., 2015), the ionic one related to the fastest and larger decay. Indeed, proton transport, which is relevant in eumelanin-based materials, is quickly blocked at the metallic contact in a dc measurement, leaving, after a transient (few seconds for EU, slightly more for EUGLs), only the “conventional” electronic one to contribute to conductivity. The following slower and less evident decay can be then ascribed to a decrease in the electronic transport, probably due to a trapping mechanism (Gargiulo et al., 2015; Wunsche et al., 2015). A simple estimation of the decaying conductivities (double exponential fit of the curves) reveals that ions contribute for more than 50% of the initial conductivity on EU, while in EUGL1, EUGL2, and EUGL3 this contribution is of the order of 20, 10, 5, respectively. It is worth noting that no time decay is observed on GL (indicating both essential electronic and “clean” transport), while on EUGL3 the “slow” decay is not observed (indicating, together with the occurrence of a residual, although strongly reduced, ion contribution, a stronger GL character in the electronic transport). For the main plot, we reported the σ_dc_ values after 900 s; furthermore. Several factors can concur to the strong improvement of σ_dc_ in the hybrids: apart from chemical modifications with related changes in the intrinsic electrical transport properties of basic units, different electrical connections in the samples (of grain or at a molecular/supramolecular level) as well as structural modifications could be responsible of the observed behavior. Spectroscopic measurements have been very helpful to shed light on this issue.

**Figure 5:**
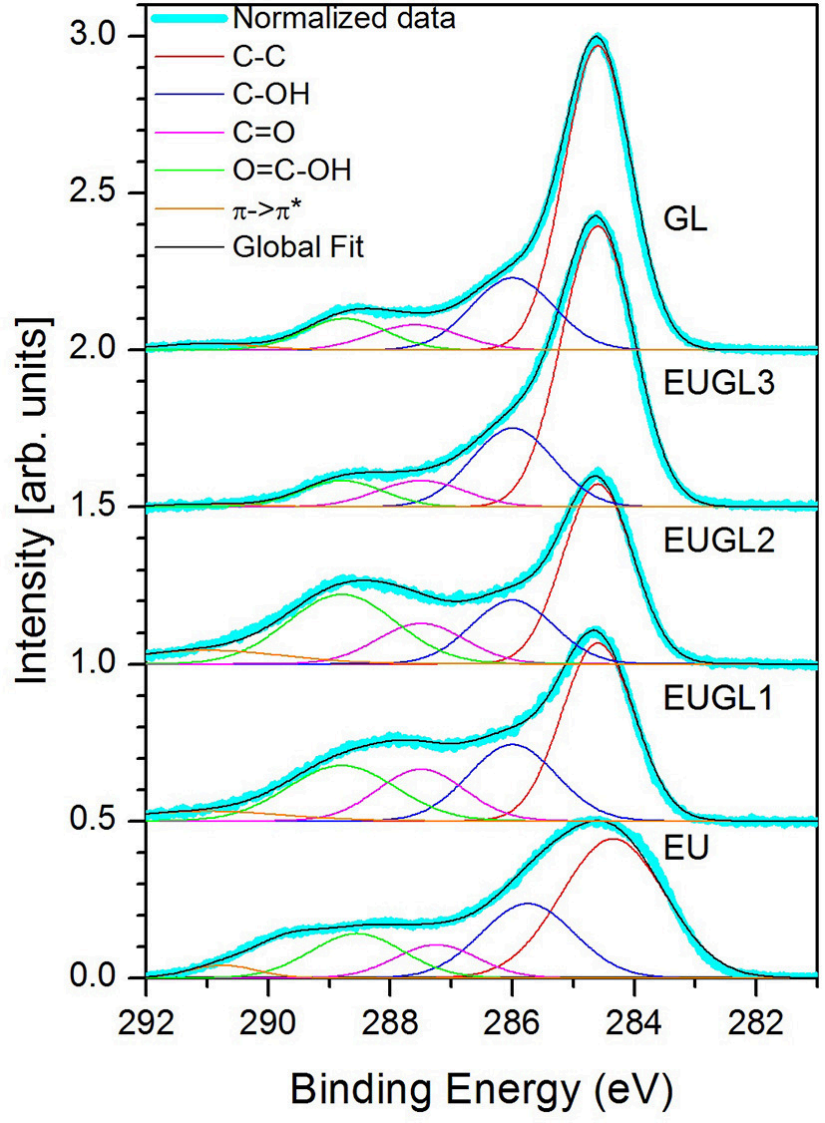
C 1s XPS measurements and related fits. Data and fits for each sample have been area normalized and vertically shifted for clarity.

First spectroscopic information came from the comparison of photoemission results among the different materials. XPS spectra of GL, EU and hybrid samples at photon energy 410 eV are reported in Figure 5. Spectra have been normalized to the same total area and Shirley background has been subtracted. Then the spectra were fitted using 5 Gaussian peaks.

The C 1s XPS spectrum of GL exhibits a very pronounced peak at binding energy of 284.6 eV, corresponding to C=C systems, with the typical asymmetric shape due to the presence of both sp^2^ and sp^3^ bonds. The second peak around 291 eV is related to the π-π^*^ satellite band of the graphitic carbon band. The fitting of the spectrum puts also in evidence peaks at 286.5 eV (C–OH from hydroxyl groups), 288.0 eV (C=O from carbonyl groups), 288.7 eV (COOH, carboxylic groups), in a relative amount already recorded on GL samples produced with our method (Alfè et al., 2015; Gargiulo et al., 2018). Compared to such spectrum, on EU sample the main C=C peak is strongly broadened and more symmetric, as expected for non-graphitic materials (Abbas et al., 2011); dominating all the other contributions (Figure 5). The photoemission spectrum collected on EUGL3, the hybrid sample with the largest GL amount, strongly resembles the GL one. On the contrary, spectra from EUGL1 and EUGL2 are more influenced by the presence of EU: they exhibit a C=C peak qualitatively similar to GL, but the other spectral contributions clearly resemble those of EU. Therefore, we can say that EUGL3 exhibits spectroscopic features close to those of the GL parent compound, while EUGL1 and EUGL2 are more affected by EU features. XPS, indeed, reveals a similar relative abundance of the different functional groups between EU, EUGL1, and EUGL2. Analogous similarities have been observed in the FTIR spectra and in the elemental compositions: such remarks resulted to be very helpful for the interpretation of the NMR results, as shown below.

The relative weights of the different spectral components (measured as the relative area of the corresponding Gaussian contribution) in the different XPS spectra are reported in Table 2.

**Table 2:** Summary of C 1s XPS spectra fits.

**Peak energy (eV)**	**Attribution**	**Relative spectral weight (%) in**				
		**EU**	**EUGL1**	**EUGL2**	**EUGL3**	**GL**
284.6	C-C and C-H	45	41	41	64	64
286.0	C-OH	23	21	17	20	19
287.6	C=O	10.9	14.3	12.5	7.3	6.9
288.8	O=C-OH	18.2	19	22.7	7.2	8.2
291.0	π-π^*^	2.9	4.7	6.8	1.5	1.9

Notably the increase of the relative weights of the C=O and COOH groups pairs with the reduction of C-OH group number in the lower graphene-loaded hybrids (EUGL1 and EUGL2).

To get further insights into the spatial arrangement of materials constituents, ss-NMR spectra have been collected on the fabricated parent and hybrid compounds, as well as on the eumelanin precursor (DHI) for comparison.

All the ^1^H MAS NMR spectra (Figure S3) are characterized by a broad signal peaked at 7 ppm, due to aromatic hydrogens of indole or pyrrole rings. The broad shape of this main signal is not only indicative of the strong dipolar coupling among hydrogen nuclei, but also of the various chemical environments around aromatic hydrogens, and of the variety of connections among various groups in the eumelanin and the related hybrids. The sharper peaks detected between 0 and 2 ppm in DHI and EU spectra are due to aliphatic impurities belonging to the synthetic procedure.

**Figure 6:**
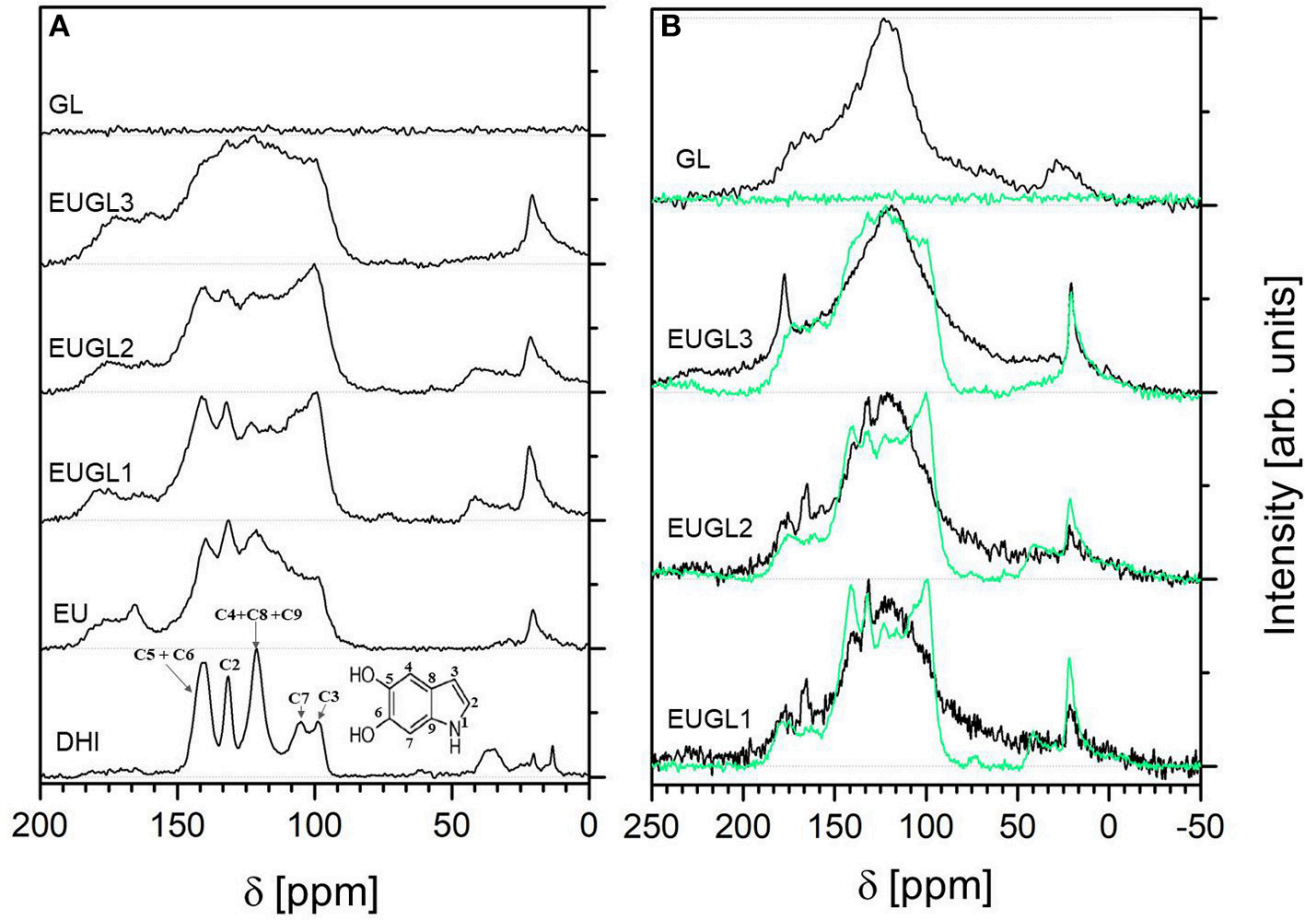
**(A)** ^1^H-^13^C CPMAS NMR spectra; **(B)** comparison between ^13^C MAS (black line) and ^1^H-^13^C CPMAS (green line) NMR spectra of GL and the three hybrids. Spectra have been height normalized and vertically shifted for clarity.

The directly excited ^13^C MAS experiments demonstrated that the spectra of all these materials are the results of a large number of overlapping resonances causing the broadening of the signals and hampering the individual carbon signals assignment (Figure 6).

To overcome this limitation and to increase the signal of carbon nuclei, ^1^H-^13^C CPMAS NMR experiments were performed. Briefly, the CP experiment relies on the proton-to-carbon polarization transfer (Pines et al., 1973). Since the efficiency of polarization transfer depends on the distances between the protons and the carbons, the CP experiment is particularly suitable to differentiate among the different classes of carbons inside a complex carbon-based network, i.e., among the protonated and the non-protonated carbons.

The ^1^H-^13^C CPMAS NMR spectrum of DHI (Figure 6A) presents a fine structure with signals directly associable to C–H carbons (C3 at 99 ppm, C7 at 106 ppm), quaternary carbons (C8, C9, and C4 at 121 ppm) and carbons bonded to heteroatoms (C5 and C6 at 141 ppm and C2 at 132 ppm) according to the assignments given for natural eumelanins (Xin et al., 2015). Signals at high magnetic field (20–40 ppm) account for residual C–H signals of those aliphatic impurities also detected in ^1^H MAS NMR spectrum. Compared to DHI, the spectral features of EU are broader and much less defined, as a consequence of the larger disorder associated to melanin polymer backbone after the oxidative polymerization has occurred. As expected, the EU spectrum contains arenes resonances consistent with the proposed indole-based aromatic structure and signals due to oxygen bearing groups in accordance with the data reported for DHI-based melanin (Hervé et al., 1994; Xiao et al., 2018) and a related material such as polydopamine (Chatterjee et al., 2014). In particular, the signals typical of H-bearing aromatic or quaternary carbons are located in the region between 90 and 140 ppm, and those typical of C–O and C=O groups in the range 170–200 ppm. An aspect deserving attention moving from the spectrum of DHI to the spectra of EU and the hybrids is the presence of signals in the carbonyl region (150–200 ppm), revealing that an increase of the oxidation level of the indoles in the backbone of the pigment takes place during the polymerization process. This finding agrees with the relative weights of the oxygen-containing functional groups estimated by XPS (Table 2).

As for EU, ^1^H-^13^C CPMAS NMR spectra of hybrid materials contain overlapping signals typical of aromatic C-H or quaternary carbons in the region between 90 and 140 ppm and signals typical of C–O and C=O groups in the range 170–200 ppm. With the increase of GL content, the fine structure of the DHI and EU spectra is lost and some significant spectra modifications reveal the actual occurrence of an intimate contact between EU and GL layers embedded in the hybrid material during the co-polymerization process. More precisely, during the EUGL hybrid synthesis the embedding of GL layers into the nascent EU polymer induces a marked modification of relative intensity ratios of carbon signals with respect to eumelanin (Figure 6B): the most striking feature of EUGLs spectra compared to EU one is the enhancement of the signals at 140 and 100 ppm with respect to those in the region around 120 ppm in EUGL1 and EUGL2 (this occurrence is less pronounced in EUGL3, whose spectral response is probably more strongly affected by GL features, consistently with the indication provided in the following by XPS). The observed signal evolution should be ascribed to an enhancement of ^1^H-^13^C cross-polarization effect for hydrogen-bonded carbons and/or hydrogen-near substituent carbons and could not be related to an actual increased relative abundance of the carbon species responsible of such signals as a consequence of the inclusion in GL, also considering the relative weakness of these feature in both parent compounds.

To confirm such idea, directly excited ^13^C MAS NMR spectra recorded on EUGL hybrids are contrasted with the corresponding ^1^H-^13^C CPMAS spectra in Figure 6B: the spectra collected in direct excitation mode are broadened and characterized by a main signal peaked at 125 ppm ascribable to aromatic carbons inside both the graphitic network of GL and the π-conjugate structure of eumelanin (protonated indole and pyrrole moieties) (Hervé et al., 1994). The lack of distinguished signals at 100 and 140 ppm in these spectra is a strong indication that the mentioned observations in CPMAS spectra on EUGLs are actually due to ^1^H-^13^C CP mechanism for selected carbon sites, and the profile evolution detected for EUGLs spectra may thus be justified on the basis of relevant intermolecular factors which governs the mutual arrangement of eumelanin components in presence of GL layers.

An added proof of this hypothesis was obtained by performing ^13^C MAS and ^1^H-^13^C CPMAS NMR experiments on a physical mixture of eumelanin and graphene like layers (EU+GL3) resembling the composition of EUGL3. This supplementary inspection aimed to check the occurrence of cross polarization effects also when EU and GL components are simply mixed. The results of the NMR experiments on this mixture are shown and compared with those of EUGL3 in Figure S4. The ^13^C MAS NMR spectrum of EU+GL3 showed a shape very similar to that of the spectrum of EUGL3; on the contrary the ^1^H-^13^C CPMAS NMR spectrum exhibited a very noisy shape overall dissimilar to that of the EUGL3. As expected, in the ^13^C MAS NMR experiment the contributes of both components of the mixture generate a spectrum with a broad signal peaked around 120 ppm in the region typical of aromatic carbons. In the case of the ^1^H-^13^C CPMAS NMR experiment the absence of a real spatial proximity between carbons and protons in EU+GL3 mixture (the EU and GL components do not truly interact) as that established in EUGL3 does not allow the instauration of a significant cross polarization effect.

Both findings concur to sustain the hypothesis of an improved eumelanin packing induced by the GL layers presence in the synthetic medium and thus during the polyindole system build-up.

By comparison with the remarks and discussion on FTIR and XPS results, we can infer, in a self-consistent scheme, that the remarked peculiar features in EUGL1 and EUGL2 CPMAS NMR spectra, compared to the EU one, should be ascribed to different arrangement of building blocks and supra-molecular units, rather than differences in the fundamental units themselves coherently with the common origin of these materials from DHI.

**Figure 7:**
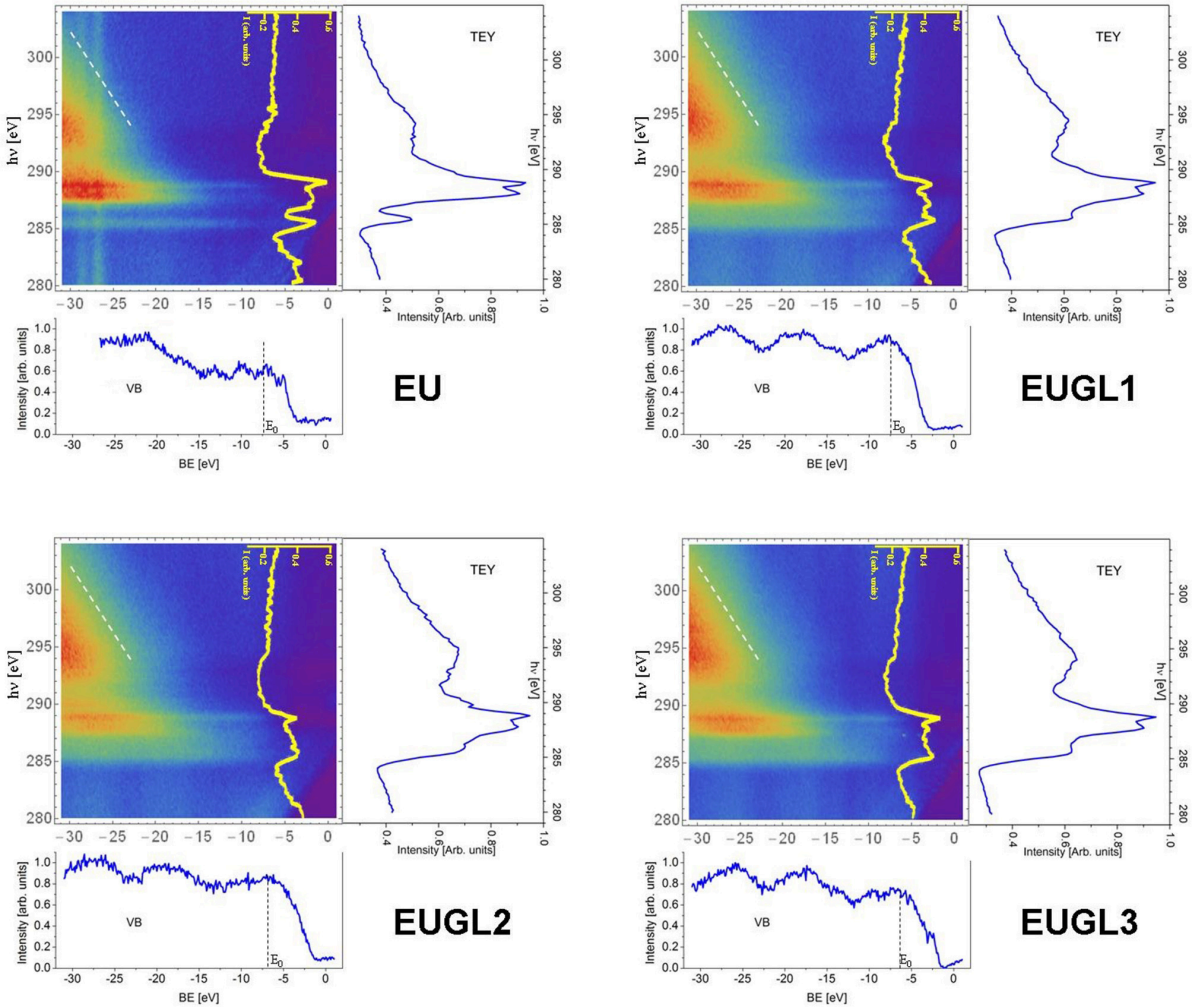
ResPES maps recorded on EU, EUGL1, EUGL2, EUGL3 reported together with the extracted valence band (below each map), XAS spectrum (on the right), and a CIS profile vs. photon energy at a given energy E_0_ corresponding to a marked feature in the valence band profile (yellow curve). The dashed white lines indicated the Auger spectroscopic features.

The difference in the microscopic mechanisms occurring in the different samples has been deeper investigated by resonant photoemission spectroscopy (ResPES). In such measurements, photon energy is set close to the absorption edge of a core level, so that the excited electron is not (directly) emitted, but it is excited in an empty valence state (resonance): the (possible) consequent decay of the excited electron produces an autoionization, and the emitted electron is detected. A ResPES map is obtained collecting the energy spectrum of the emitted photoelectrons at several photon energies around the resonant edge. In Figure 7 we report valence band (VB) ResPES plots for EU and for the three EUGL hybrids, as maps of photoemission intensity as a function of photon excitation energy (hν) and of binding energy (BE) of the photoelectron initial state. The photon energy is scanned from 280 to 304 eV across the C 1s absorption threshold, producing resonant excitations of such core electrons toward the first unoccupied orbitals. Total electron yield (TEY) mode X-ray absorption spectrum obtained by integrating ResPES intensity along the binding energy, is shown on the right of each ResPES map, all having a common photon energy scale. The horizontal photoemission profile at hν = 281 eV (below the resonances) is extracted and reported below each ResPES map (on a common binding energy scale), to highlight the main spectral features of the VB of the samples.

For sake of clarity the ResPES map and the extracted curves of GL are reported in Figure S5.

The observed main features, as concerns both EU and EUGL samples, are similar to what was already reported on commercial melanin powders (Sangaletti et al., 2009; Borghetti et al., 2012). The spectral features in the valence band at BE from 0 to about −10 eV are mainly due to N 2p, O 2p, and C 2p orbitals; then, up to BE of about −20 eV there are contributions from σ orbitals, while the features at deeper binding energies come from core levels of oxygen, nitrogen, carbon atoms. In the ResPES map, the most evident features are the Auger emission (dashed white line) with its characteristic dispersive behavior vs. BE, and the resonance detected around the photon energy of the first x-ray absorption peak (excitations toward π^*^ empty orbitals typical of aromatic rings). This resonance occurs when the electron excitation is toward a bound empty level, followed by a core-hole autoionization decay: the final state is a two (core) hole-one (excited) electron state, shifted at higher BE compared to the normal Auger final state (a two-hole state).

On a ResPES intensity map, a constant initial state (CIS) profile, i.e., intensity profiles vs. hν at a fixed BE, can provide information on the charge transfer dynamics. In Figure 7 we extracted the CIS profile corresponding to BE = E_0_ for each presented ResPES map (E_0_ being the BE value of a resonance highlighted in each VB profile). The intensity profiles enhance (resonate) at hν around 286 and 289 eV. These are the photon energies of the first two XAS resonances, corresponding to excitations into π^*^ and σ^*^ orbitals, respectively. The resonant Auger emission can take place as long as the excited electron remains localized for a time long enough (typically at least comparable to the core-hole lifetime) in order to allow the autoionization decay process. If the excited electron, instead, “quickly” delocalizes, a final state identical to normal Auger emissions happens, therefore not contributing to the resonance in CIS profile. The intensity of the resonance is therefore proportional to the probability that de-excitation occurs before the delocalization of the electron excited into a bound state, which in turn is directly proportional to the delocalization characteristic time. Then, a CIS resonance lacking or weakened indicates a “faster” delocalization, i.e., a stronger interaction with the surrounding material. From the CIS profiles at BE = EB in Figure 5, it is evident how the resonance at hν corresponding to XAS peaks is clearly less pronounced for EUGL1 and EUGL2 compared to EU (this is true also of EUGL3, even if less evident: a further similarity between EUGL3 and GL, which also exhibits quite pronounced resonances). For the discussion above, this occurrence indicates a faster delocalization mechanism in the hybrids, which represents another indication of a tighter packing and/or a stronger interaction between single units as a consequence of the inclusion of GL layers.

**Figure 8:**
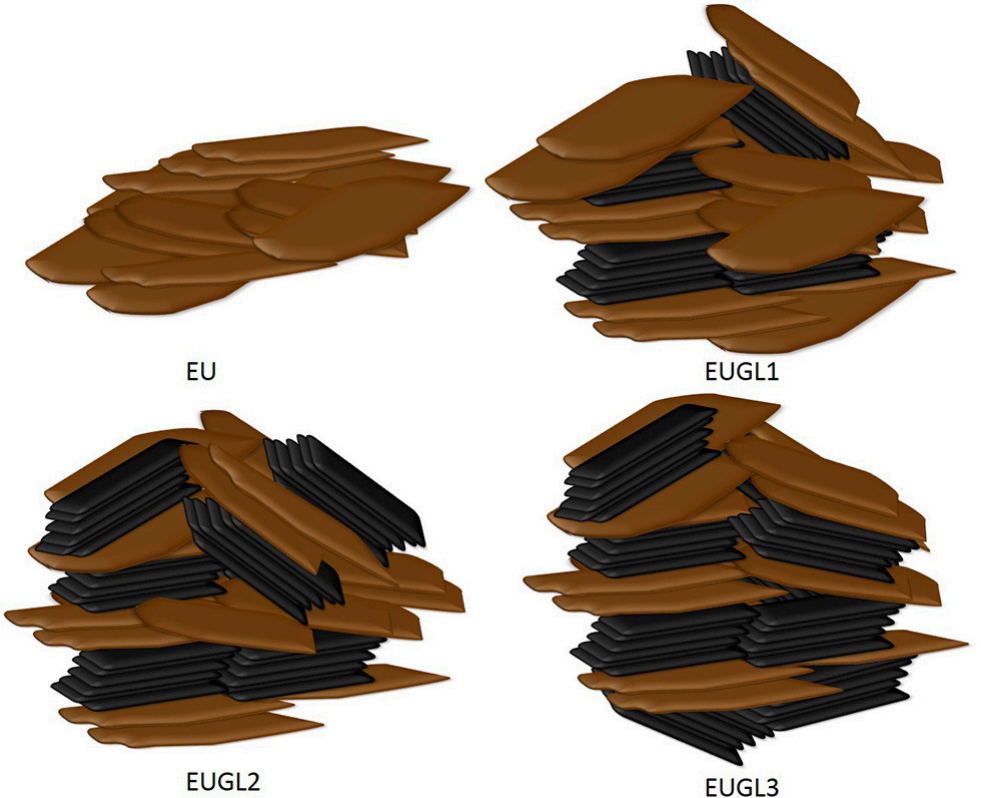
Pictorial description of EU and EUGLx hybrids.

Several experimental findings from different techniques are therefore agreeing as concerns the occurrence of a stronger packing in the hybrid samples compared to pure eumelanin. From a structural point of view, the most obvious consequence is a reduction of spacing between elementary units and of π-π stacking distance; likely, it also could correspond to a more ordered structure (a point, however, beyond the scope of the present work and deserving deeper investigation), despite the realization of more complex compounds whose pictorial description is proposed in Figure 8.

These circumstances can actively contribute to the observed enhancement of electrical dc-conductivity (Figure 4). In organic semiconductors, indeed, the distance between π-conjugated oligomers and polymers strongly affects the overlapping between adjacent orbitals, and therefore the amplitude of the electronic transfer integrals between the various interacting units (Brédas et al., 2002). The mobility of charge carriers is increased by an enhancement of the transfer integral, as a consequence of a more efficient hopping between neighbors (Brédas et al., 2002); experimental evidences of such effects have been provided, for examples, for TIPS-pentacene (Giri et al., 2011) (a reduction of 8% in π-π stacking distance leaded to a six times increase of hole mobility), conjugated polymers (Ma et al., 2016) (significant enhancement of electrical conductivity obtained as a consequence of better ordered structure and molecular packing), conductive polymer/carbon nanotube hybrids (Kim et al., 2016) (a dense close packing of nanotubes cores and polymer shells increased the electrical conductivity almost 20 times). Tighter packing and possibly more ordered structure can positively affect the electrical conductivity even in the case of electrical transport driven by percolation mechanism (Sahimi, 1994).

## Conclusions

We investigated different samples prepared by *in situ* polymerization of eumelanin precursor DHI in presence of various amounts of GL layers. Basic characterizations, together with NMR and photoemission studies, provided information about the correlation between structural/spectroscopical rearrangement and the electrical properties as a function of the relative composition. Solid state NMR data indicated that structural and packing modification occur in the hybrid compounds with respect to eumelanin: in particular, the observed enhanced cross-polarization between ^1^H and ^13^C demonstrates a stronger synergic interaction of the two materials to gain increased fill factor, which is likely associated to the higher electrical conductivity. ResPES measurements support this interpretation, giving evidence of a stronger interaction, in the hybrids, of the single atom with the surrounding environment through the observation of a faster delocalization mechanism. It is worth to note, however, that in the richest GL hybrid among those investigated, the GL-character starts to become prevalent, as also confirmed by other chemical and physical characterizations. With this work, we performed a first study on the fundamental mechanisms occurring when eumelanin is integrated with graphene-related conducting materials, and responsible of the main observed macroscopic properties (electrical conductivity, biocompatibility, adhesion…), which represent an important knowledge in order to make prediction of results when synthesizing hybrid structures.

## Data Availability

All datasets generated for this study are included in the manuscript and/or the supplementary files.

## Author Contributions

AP, RDC, MA, and VG contributed to the conception and design of the study. AP, MA, and VG synthetized the materials and their precursors. VG and MA performed elemental analysis, FTIR spectroscopy and thermogravimetric analysis. RDC and GMDL performed electrical material characterization. AP, MA, VG, and GM contributed to ss-NMR data acquisition. RDC, MA, VG, and TS contributed to spectroscopical data acquisition with the use of synchrotron radiation and their elaboration. All authors contributed to the interpretation and the analysis of the data. All the authors wrote sections of the manuscript, contributed to manuscript revision, read, and approved the submitted version.

### Conflict of Interest Statement

The authors declare that the research was conducted in the absence of any commercial or financial relationships that could be construed as a potential conflict of interest.
