# Supplementary material for: Eumelanin Graphene-Like Integration: The Impact on Physical Properties and Electrical Conductivity
Source: Front Chem. 2019 Mar 18;7:121. doi: 10.3389/fchem.2019.00121 (PMC6432792; doi:10.3389/fchem.2019.00121)
Supplement: Supplementary file 1 [file Data_Sheet_1.pdf]

Supplementary Material

Eumelanin Graphene-Like Integration: The Impact on Physical Properties and Electrical Conductivity

Roberto Di Capua, Valentina Gargiulo, Michela Alfè, Gabriella Maria De Luca, Tomáš Skála, Gregor Mali, Alessandro Pezzella*

*** Correspondence:** alessandro.pezzella@unina.it

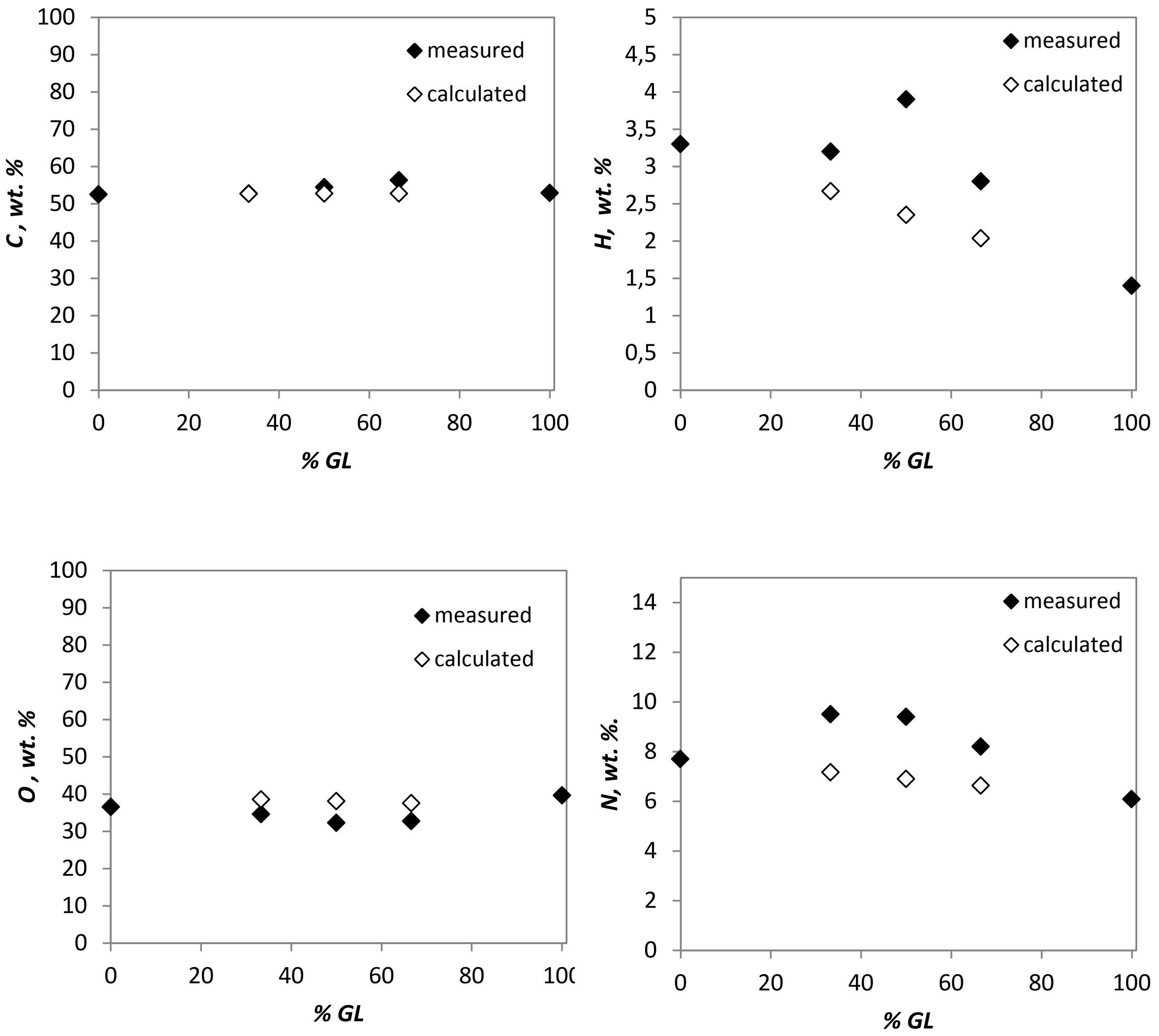

Figure S1. Comparison between theoretical and measured C, H, N and O contents.

The “theoretical” percentages value of each element content was calculated according with the following equation:

$X_{tot} = X_{GL} \text{ x } f_{GL} + X_{EU} \text{ x } f_{EU}$

where X_{tot} (X = C, O, H or N) is the element percentage in the composite (expressed as wt. %), X_{GL} and X_{EU} are the percentage (wt. %) of the same element in GL and EU respectively, and fGL and f_{EU} are the weight fractions of GL and EU in the composite ($f_{GL} = 1 – f_{EU}$).

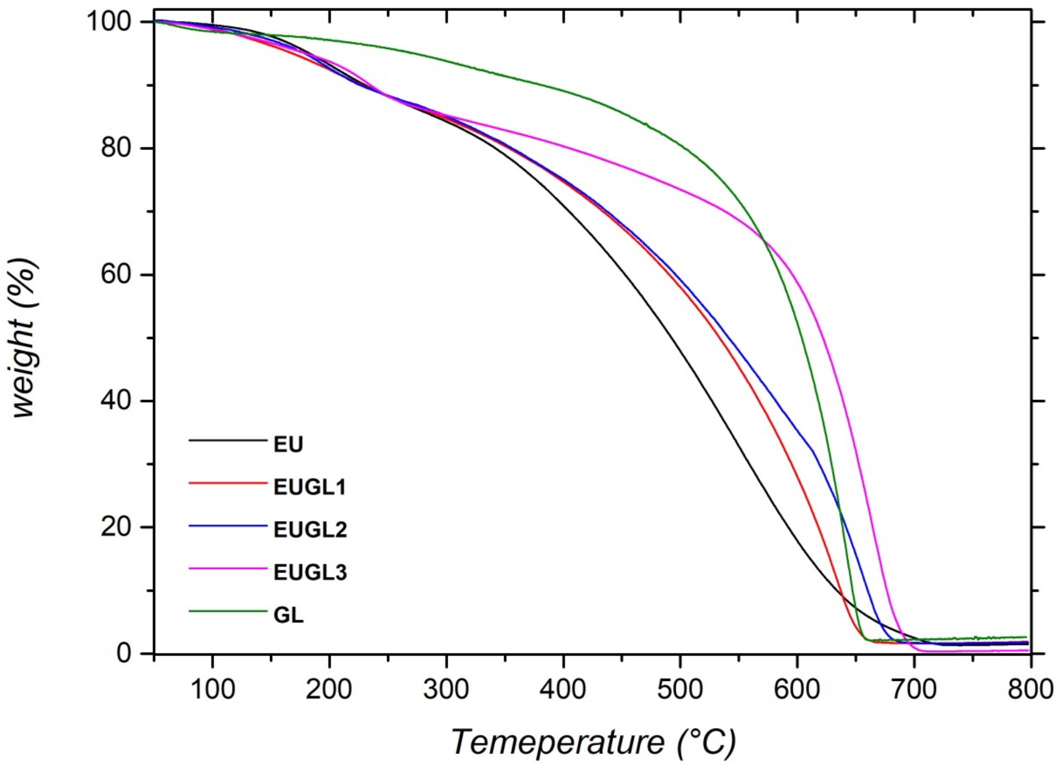

Figure S2. Thermogravimetric profiles of the EU, GL and the three hybrid materials.

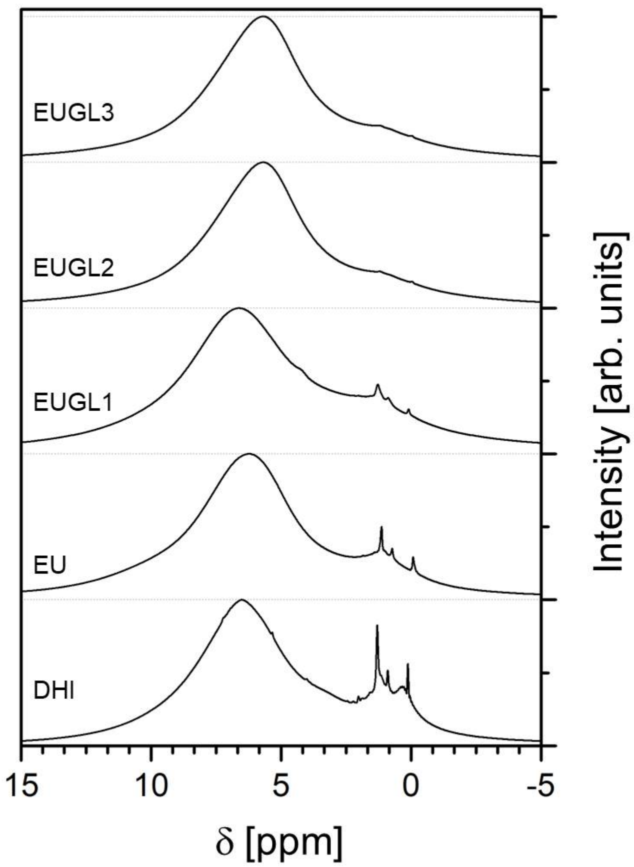

Figure S3.^{1}H MAS NMR spectra

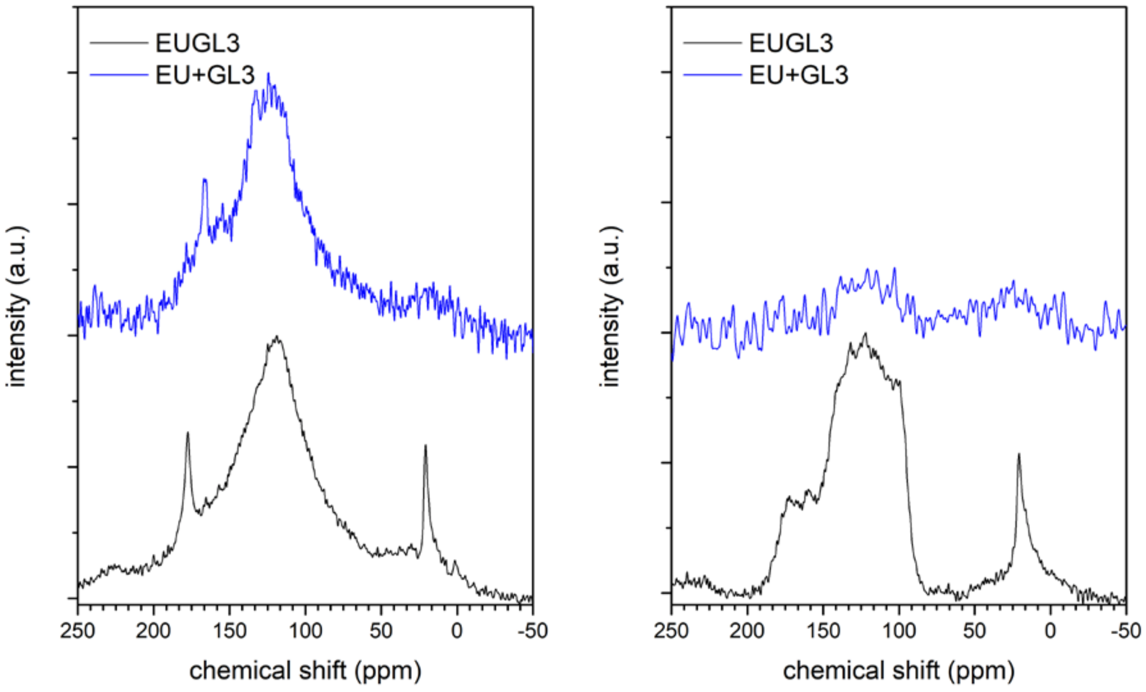

Figure S4. Left panel: comparison between ^{13}C MAS NMR spectra of EUGL3 and the physical mixture EU+GL3; right panel: ^{1}H-^{13}C CPMAS NMR spectra of EUGL3 and the physical mixture EU+GL3

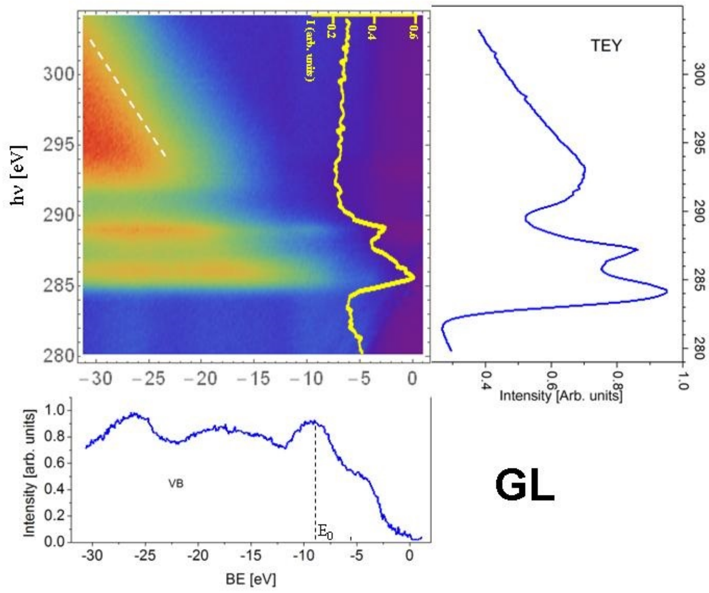

Figure S5. ResPES maps recorded on GL reported together which the extracted valence band (below the map), XAS spectrum (on the right), and a CIS profile vs. photon energy at a given energy E0 corresponding to a marked feature in the valence band profile (yellow curve).
